# Virtual Reality-Based Attention Bias Modification Training for Social Anxiety: A Feasibility and Proof of Concept Study

**DOI:** 10.3389/fpsyt.2015.00154

**Published:** 2015-10-28

**Authors:** Antoine Urech, Tobias Krieger, Alvin Chesham, Fred W. Mast, Thomas Berger

**Affiliations:** ^1^Department of Psychology, University of Bern, Bern, Switzerland

**Keywords:** attention bias modification, dot-probe paradigm, attention bias, virtual reality, social anxiety disorders, social phobia

## Abstract

Attention bias modification (ABM) programs have been considered as a promising new approach for the treatment of various disorders, including social anxiety disorder (SAD). However, previous studies yielded ambiguous results regarding the efficacy of ABM in SAD. The present proof-of-concept study investigates the feasibility of a newly developed virtual reality (VR)-based dot-probe training paradigm. It was designed to facilitate attentional disengagement from threatening stimuli in socially anxious individuals (*N* = 15). The following outcomes were examined: (a) self-reports of enjoyment, motivation, flow, and presence; (b) attentional bias for social stimuli; and (c) social anxiety symptoms. Results showed that ABM training is associated with high scores in enjoyment, motivation, flow, and presence. Furthermore, significant improvements in terms of attention bias and social anxiety symptoms were observed from pre- to follow-up assessment. The study suggests that VR is a feasible and presumably a promising new medium for ABM trainings. Controlled studies will need to be carried out.

## Introduction

Social anxiety disorder (SAD) is characterized by an intense fear of being criticized, judged, or rejected by others (1). SAD ranges among the most common mental disorders, with an estimated lifetime prevalence of 12.1% (2) and leads to personal, economic, and societal costs as well as comorbidity with other disorders (e.g., depression) (3).

Cognitive models of SAD suggest that socially anxious individuals are prone to biases at specific stages of information processing (4). In SAD, the attentional system is abnormally sensitive to threat-related stimuli, and affected individuals tend to direct their attention toward threatening information during early, automatic stages of processing (5). Accordingly, reflecting the proposed hypervigilant mode toward threat in SAD, a meta-analysis showed that anxious individuals detect threat-related stimuli significantly faster than neutral ones (6). In contrast, alternative models highlight the avoidance mechanism and posit that threatening information is avoided or inhibited (7), and that anxiety has less impact on the initial detection of threat, but rather a stronger effect in modulating the maintenance of attention on the source of threat (8). Furthermore, individuals suffering from SAD showed prolonged disengagement from threat (9). In summary, there is evidence that social anxious individuals differ from non-anxious individuals in their attention regarding their detection, disengagement, and avoidance of social threat information.

As a consequence, attention bias modification (ABM) studies have emerged to modify the attention bias and thus reducing anxiety in SAD (6, 10). ABM trainings aim at directly modifying the attentional system and patterns of neural activation in social anxious individuals in the context of the dot-probe paradigm (10, 11). The majority of attention trainings manipulated the attention bias away from threat and onto a neutral stimulus, because this approach proved efficient in some clinical trials (4, 12, 13). However, it is still not clear whether this procedure is indeed the most potent approach available (10).

Findings from different ABM training studies remained inconclusive (13–18). One possibility is that the lack of ecological validity and incomplete immersion impeded the success of some of the earlier studies, e.g., due to the fact that all ABM trainings were conducted on desktop computers or smartphones. Besides the lack of ecological validity, the use of multiple experimental manipulations in different studies (e.g., presentation length, stimulus type, or the study population) may have led to mixed results (15). Here, we propose the use of virtual reality (VR) in ABM training.

Virtual reality applications are defined by allowing the user to navigate through and interact with an environment that is close to its natural counterpart (19–21). VR enables to perform real physical actions (e.g., motor tasks) and the manipulation of virtual objects (22). Such enactments of bodily movements in VR might strengthen approach behaviors, which have been found to be crucial in SAD (23, 24). Furthermore, these possibilities lead to an improvement of the user experience compared to other media (e.g., desktop computers) (22). Moreover, it has been postulated that a high level of presence is positively associated with task performance (25), enjoyment (26), flow (27), and motivation (28). In general, it has been found that VR elicits stronger ratings on presence than desktop computers (29). To date, VR has successfully been adapted to exposure therapy, and studies have repeatedly found good long-term follow-up for several anxiety disorders (30–33). Consequently, the use of VR may have several advantages in ABM trainings.

The main goal of this study was to test the feasibility of a VR-based modified dot-probe paradigm in students with increased social anxiety.

## Methods

### Participants

Fifteen undergraduate students (12 females and 3 males) between 19 and 24 years (*M* = 20.2 years, SD = 1.42) with normal or corrected-to-normal vision participated in this study. All participants were recruited through advertising at the University of Bern (e.g., pin board and lectures). The study was explicitly advertised for students suffering from increased social anxiety. For their participation, they received course credits. All participants provided written informed consent before the inclusion in the present study. The study was approved by the Local Ethics Committee of the University of Bern.

### Apparatus

The VR-based modified dot-probe task was designed and rendered using the Python/OpenGL-based VR toolkit Vizard (WorldViz LLC, Santa Barbara, CA, USA). The virtual environment (VE) was modeled and textured using the open-source three-dimensional (3D) graphic software Blender (Blender Foundation, 2013). Participants were wearing a stereoscopic nVisor SX60 head-mounted display, which rendered the VE at 1,280 × 1,024 resolutions with a 60° diagonal field of view for each eye. The participant’s position and body movement was tracked using the Microsoft Kinect Xbox 360, 250 GB (Microsoft, Redmond, USA). The whole body tracking device’s depth-camera (Microsoft Kinect) was calibrated using the Flexible Action and Articulated Skeleton Toolkit (FAAST). The FAAST driver is an interface allowing for streaming the participant’s skeleton to the VR engine over a VR peripheral network (VRPN) server.

### Intervention

We created a VR-based modified version of the dot-probe task used in the previous studies to change the attention bias (15). The aim of this intervention was to associate a probe to a neutral cue, hence turning the attention away from the simultaneously presented negative cue.

**Figure 1:**
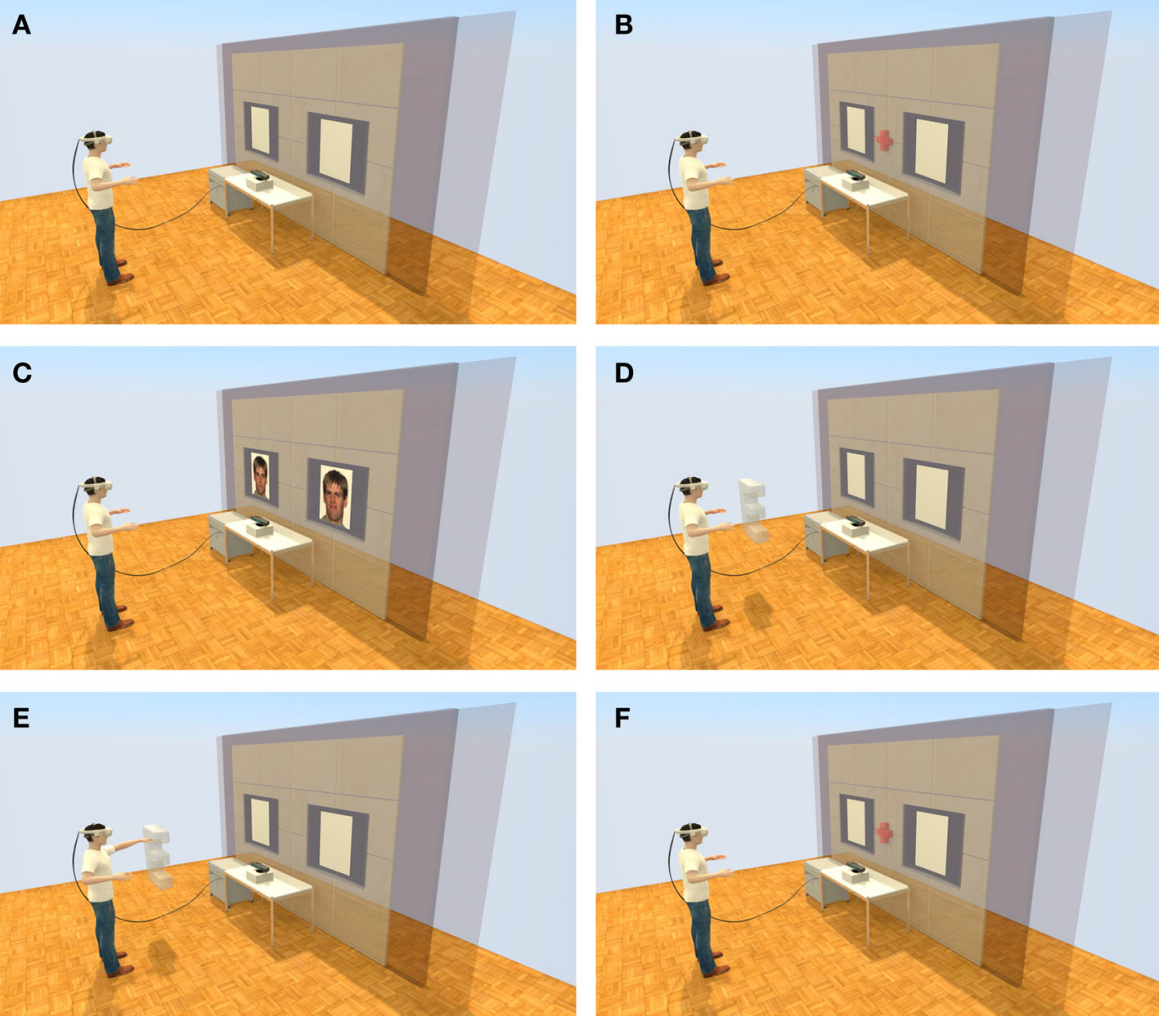
**Schematic of the VR setup**. The semitransparent part (i.e., the wall with the two blank picture frames) of the image represents the VE the participants viewed. **(A)** A Microsoft Kinect was used as a whole body-tracking device; **(B)** Illustration of the red fixation cross, which disappeared after 500 ms; **(C)** 500 ms presentation of the two face stimuli (e.g., neutral and disgust expression); **(D)** Virtual 3D letter appeared in front of the neutral face; **(E)** The participant hit the virtual letter on the left side with his left arm; and **(F)** End of trial, next trail begins with the red fixation cross again.

Each dot-probe trial began with a fixation cross (+) presented in the center of a 3D model of a video conference wall for 500 ms. Directly after the fixation cross, two faces of the same individual were presented for 500 ms in two 3D picture frames on the 3D video conference wall, one on the left-hand side and one on the right-hand side. We used face stimuli from the NimStim set (34) and selected faces of eight individuals (four male and four female). After presenting the faces, a 3D model of a letter (E or F) appeared in front of the location of one of the faces. Participants were asked to hit the letter with their arms. If a 3D letter was presented on the left side, participants were told to hit the 3D letter with their left hand and vice versa for letters on the right-hand side. After the virtual hand and the 3D letter collided, the next trial began. Figure 1 shows an example trial of the VR-based modified dot-probe task. The instruction was to react (by hitting the letter) as quickly and accurately as possible. Before the actual dot-probe training session started, participants completed practice trials with pictures of fruits and houses instead of faces. The training session consisted of 160 dot-probe trials. The distance between the tracking system and the participants was kept constant (1.5 m) with a mark on the floor. The distance was based on the recommendations of Microsoft to achieve optimal resolution (e.g., range between 1.2 and 3.5 m). Participants were instructed not to move their feet during the experiment.

Furthermore, the VE as well as the NimStim set was preloaded in the cache to avoid further latencies. Typically, latency times are 106 ms for the Microsoft Kinect (35) and 5 ms for the VRPN server.

### Self-Report Measures

Upon completion of the training, participants filled out the flow questionnaire (36) (example: “I feel perfectly claimed” one = disagree; seven = agree) and the eight-item presence scale (37) (example: “the VR-training created a new world for me, which immediately disappeared when the training ended” one = not at all; seven = very strong). Additionally, they answered a single item to assess motivation (“How motivated were you to play the VR-training?” one = not at all; five = very much) and enjoyment (“Did you enjoy the VR-training?” one = not at all; five = very much) (38, 39). For measuring social anxiety symptoms, we used the following scales: the Liebowitz Social Anxiety Scale (LSAS-SR) (40), the Social Phobia Scale (SPS), and the Social Interaction Anxiety Scale (SIAS) (41).

### Attention Bias Assessment

We used a modified version of the Posner task (42, 43). In order not to assess stimulus specific effects, we used words rather than faces as stimuli in the attention bias assessment. Similar to a previous study (15), we chose eight social threat words (e.g., “embarrassed”) and eight neutral words (e.g., “original”) out of a standardized set of words (44). The presented words were matched for length and frequency in the German language. The modified Posner task began with the presentation of a fixation cross, which was centered between two rectangles. After the fixation cross, a neutral or a social threat word was displayed in one of the two rectangles (left: −36.87°; right: +36.87°) for 600 ms. Following the presentation of the word, a cue (*) appeared either at the location of the word (valid trial) or opposite of it (invalid trial). Participants were instructed to react to the cue as quickly as possible. The attention bias assessment consisted of 192 trials (128 trials valid trials, 32 invalid trials, and 32 uncued trials). The attention bias was assessed at pre-, post-, and follow-up assessment. The individual bias score was calculated by subtracting reaction times of invalid social threat trials from reaction times of invalid neutral trials. The greater the absolute value of this bias score, the more pronounced the bias (15). We eliminated 1.34% of the trials because of response latencies <50 ms or >1,200 ms (13).

### Procedure

All participants read a standardized information and instruction sheet. After this, the participants filled out the SAD outcome measures online, followed by the assessment of the attention bias. Then the participants started the VR-based modified dot-probe task. The training took about 10 min. Afterwards, the self-report measures (presence, motivation, flow, and enjoyment) were administered in a paper–pencil version. After the training, attention bias was assessed a second time. Six weeks after the training session, participants were invited by e-mail to complete the whole assessment online (follow-up).

### Statistical Analyses

Data were analyzed with SPSS 21. All data were analyzed for normality using the Kolmogorov–Smirnov test as well as the Shapiro–Wilk test; all tests were not significant, indicating normal distribution. Furthermore, all analyses were calculated based on the completed sample (14/15 participated in the postassessment and 13/15 in the follow-up assessment). In addition to the descriptive analyses, dependent *t-*tests (one tailed) were conducted to examine changes in the attention bias and SAD outcome measures. Additionally, the relationship between the process measures and social anxiety residualized gain scores (pre–follow-up measurements controlled for premeasurements) was calculated based on Spearman correlations.

## Results

Descriptive analyses showed that participants rated flow (*M* = 4.87; SD = 1.11), motivation (*M* = 4.13; SD = 0.63), presence (*M* = 3.46; SD = 0.96), and enjoyment (*M* = 3.79; SD = 0.79) above the midpoints of the scales. These ratings are relatively high when compared to the previous studies using the same scales (38, 39). There was no significant change in attention bias from pre to post (*P* = 0.132). Interestingly, however, there was a significant decrease of the attention bias from pretest to follow-up (*P* = 0.026).

From pre- to follow-up assessment, dependent *t*-tests showed a significant reduction of social anxiety measured with the LSAS (*P* = 0.04) and marginally significant results for the SPS score (*P* = 0.07) and the SIAS score (*P* = 0.06) (see Table 1). However, the observed statistical significant results would not be significant, if corrected for multiple testing.

**Table 1:** **Means and SDs for social anxiety measures**.

	Mean	SD	*t*	*Df*	*P*
**Social phobia scale**					
Pre	14.20	9.88	1.57	13	0.07
Follow-up	11.21	9.61			
**Social interaction anxiety scale**					
Pre	25.20	10.42	1.65	13	0.06
Follow-up	23.21	8.93			
**Liebowitz social anxiety scale**					
Pre	44.73	21.76	1.87	13	0.04*
Follow-up	40.00	21.32			

*Pre: N = 15 and Follow-up: N = 14*.

*P < 0.05.

The relationship between social anxiety residualized gain scores on the SPS score and presence showed a significant negative correlation (*r* = −0.534, *P* = 0.049), all other correlations were not significant.

## Discussion

The aim of this study was to test the feasibility of using ABM training via VR in a single session as a new approach for individuals with social anxiety symptoms. Most importantly, the present study shows that ABM training can be implemented successfully in VR. Furthermore, with respect to the technical side of the VR-based ABM training, it is possible to track an arm movement in real time without expansive motion tracking systems.

It is noteworthy that all participants finished the VR-based ABM training and that the training was associated with high scores in enjoyment, flow, presence, and motivation, indicating good acceptance and feasibility of the intervention. Despite the short duration of the training (10 min), the attention bias and scores on the LSAS decreased significantly from preassessment to the 6 weeks follow-up assessment. Boettcher et al. (15) showed no change in attention bias over time in social anxious individuals using the same assessment procedure, as described in this study, in combination with a placebo control dot-probe training. ABM training in our study targeted the modification of reaction times to social threat stimuli. In this respect, our data indicate that reaction times to social threat stimuli changed significantly from pre- to follow-up assessment, whereas reaction times in trials with neutral stimuli did not change over time. This suggests that the present VR training showed specific effects on the target variables in the expected direction.

However, the results of the present study should be interpreted with caution. This feasibility study included no control group. Furthermore, the sample in the present study consisted of students with self-assessed increased social anxiety. Nevertheless, several authors showed that threat-related bias was similar in clinically anxious and non-clinical high-anxious participants (45, 46).

To evaluate the efficacy of the intervention, a sufficiently powered randomized controlled trial is needed. Moreover, future studies should examine whether ABM trainings could be used either as a stand-alone treatment or in combination with psychotherapy. In addition, it should be considered that as a first step to implement ABM trainings into VR, we used two-dimensional pictures of faces. More ecologically valid and realistic stimuli such as 3D stimuli of faces or meeting autonomous virtual characters in VR might improve the outcome.

In conclusion, the current study demonstrated the feasibility of a complex (e.g., body tracking and motor task) VR-based ABM training.

## Conflict of Interest Statement

The authors declare that the research was conducted in the absence of any commercial or financial relationships that could be construed as a potential conflict of interest.
